# Antibacterial Bicyclic Fatty Acids from a Korean Colonial Tunicate *Didemnum* sp.

**DOI:** 10.3390/md19090521

**Published:** 2021-09-16

**Authors:** Hiyoung Kim, Tae Gu Lee, Inho Yang, Weihong Wang, Jungwook Chin, Jusung Lee, Boon Jo Rho, Hyukjae Choi, Sang-Jip Nam, Dongyup Hahn, Heonjoong Kang

**Affiliations:** 1 Laboratory of Marine Drugs, School of Earth and Environmental Sciences, Seoul National University, NS-80, Seoul 08826, Korea; reihyoung@konkuk.ac.kr (H.K.); pharmacy2007@naver.com (W.W.); leejusung@snu.ac.kr (J.L.); 2 Department of Biomedical Science and Engineering, Konkuk University, Seoul 05029, Korea; 3 Safety Research Team, Crop Protection Research Institute, FarmHannong Co., Ltd., Nonsan 33010, Korea; leetg@farmhannong.com; 4 Department of Convergence Study on the Ocean Science and Technology, Korea Maritime and Ocean University, Busan 49112, Korea; ihyang@kmou.ac.kr; 5 Research Institute of Oceanography, Seoul National University, Seoul 08826, Korea; 6 New Drug Development Center, Daegu-Gyeongbuk Medical Innovation Foundation, Daegu 41061, Korea; jwchin@dgmif.re.kr; 7 Natural History Museum, Ewha Womans University, Seoul 03760, Korea; nhm@ewha.ac.kr; 8 College of Pharmacy, Yeungnam University, Gyeongsan 38541, Korea; h5choi@yu.ac.kr; 9 Department of Chemistry and Nano Science, Ewha Womans University, Seoul 03760, Korea; sjnam@ewha.ac.kr; 10 School of Food Science and Biotechnology & Department of Integrative Biology, Kyungpook National University, Daegu 41566, Korea; 11 Interdisciplinary Graduate Program in Genetic Engineering, Seoul National University, NS-80, Seoul 08826, Korea

**Keywords:** *Didemnum*, antibacterial, colonial tunicate, bicyclic fatty acids

## Abstract

Five new bicyclic carboxylic acids were obtained by antibacterial activity-guided isolation from a Korean colonial tunicate *Didemnum* sp. Their structures were elucidated by the interpretation of NMR, MS and CD spectroscopic data. They all belong to the class of aplidic acids. Three of them were amide derivatives (**1**–**3**), and the other two were dicarboxylic derivatives (**4** and **5**). The absolute configurations were determined by a bisignate pattern of CD spectroscopy, which revealed that the absolute configurations of amides were opposite to those of dicarboxylates at every stereogenic centers. Compound **2** exhibited the most potent antibacterial activity (MIC, 2 μg/mL).

## 1. Introduction

Marine tunicates have been proven to be rich sources of biologically active secondary metabolites. On the basis of the rationale that tunicates are likely to require chemical defense for survival as soft-bodied benthic invertebrates, they were investigated as sources of bioactive natural products [1]. Tunicates embrace diverse symbiotic bacteria, and they have been identified to be responsible for producing most of the natural products of tunicates [1]. The diversity of natural products isolated from tunicates is attributed to their frequent hosting of bacterial symbionts [2,3,4,5,6]. These metabolites are known to exhibit antibacterial [7,8,9], antiviral [10,11], antitumor [11,12,13,14,15,16,17,18,19], and farnesoid X receptor (FXR) antagonistic activities [20]. Tunicates have yielded two natural product-derived drugs, trabectedin [19] and plitidepsin (dehydrodidemnin B) [21], which has been used as an anticancer drug. However, marine tunicates are also promising sources for antibiotic natural products. As secondary metabolites are considered to be produced for survival of the producers, symbiotic bacteria in tunicates would yield diverse antibiotics for their dominance in hosts and space in marine benthic ecosystems.

Among the families belonging to tunicates (ascidians), Didemnidae is the largest family, which includes the genus *Didemnum* [22]. The genus *Didemnum* stands out of the species in the family Didemnidae, as it was described more than any other genera [23]. The genus has been recognized as one of the most interesting sources of bioactive secondary metabolites among tunicates. It harbors many different symbiotic bacteria, which are sometimes responsible for producing the bioactive secondary metabolites isolated from the host animals [24]. As a potential source of antibacterial metabolites, we kept investigating on the genus *Didemnum.* In the previous study, we described the novel alkaloids with antibacterial activity against Gram-positive bacteria from a Korean colonial tunicate *Didemnum* sp [8]. A continuous intensive study led to five new enantiomeric polyketides with hexahydro indene moiety, which is very rare in nature. Herein, we report the isolation, structure elucidation and antibacterial activity evaluation of these marine natural products from *Didemnum* sp. collected in South Korea.

## 2. Results and Discussion

### 2.1. Structure Elucidation of Compounds ***1***–***5***

**Figure 1:**
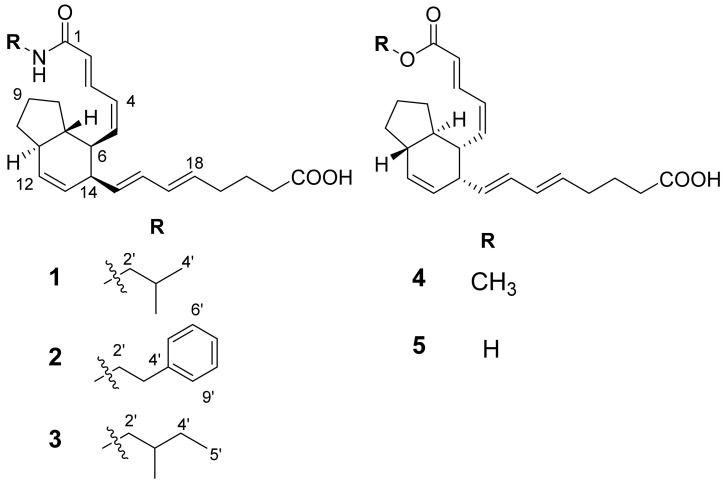
The structures of compounds **1**–**5**.

The planar structures of compounds **1**–**5** were elucidated from analysis of spectroscopic data acquired from mass spectrometer (MS) and NMR spectrometer (1D (^1^H and ^13^C) and 2D (COSY, HSQC, and HMBC) spectroscopy) (Supplementary Materials). The relative configurations were determined from analysis of NOESY spectra. The absolute configurations were determined from analysis of circular dichroism data of **1**–**5**. The structures of compounds **1**–**5** were determined as shown in Figure 1. The detailed structure elucidation process was described as follows.

#### 2.1.1. Compound **1**

The compound **1** was isolated as yellowish oil. The molecular formula of **1** was established as C_26_H_37_O_3_N on the basis of the HRFABMS data of the [M + H]^+^ ion at *m*/*z* 412.2850 (Δ +0.4 mmu), requiring nine degrees of unsaturation. The ^1^H NMR spectrum showed ten olefinic protons [δ 7.55, 6.17, 6.04, 6.02, 5.95, 5.93, 5.71, 5.56, 5.55 and 5.47], two methyls [δ 0.92 (Me × 2)] in addition to sp^3^ methines and methylenes resonating between δ 3.08 and 1.07. The ^13^C NMR spectrum exhibited 25 distinguished signals, which indicated two carbon signals were overlapped (Table 1). The analyses of ^13^C and HSQC spectra revealed two overlapped doublet methyl protons (δ 0.92, *J* = 7.0 Hz), five sp^3^ methines and seven sp^3^ methylenes. In addition, two carbonyls (δ 177.7 and 169.2) and ten olefinic carbons (δ 143.0, 137.0, 133.5, 133.2, 133.0, 132.3, 131.0, 130.9, 127.5 and 127.4) consisting of five carbon–carbon double bonds were present, which accounted for seven of, overall, nine degrees of unsaturation, thus indicating the presence of two ring systems in the molecule.

**Figure 2:**
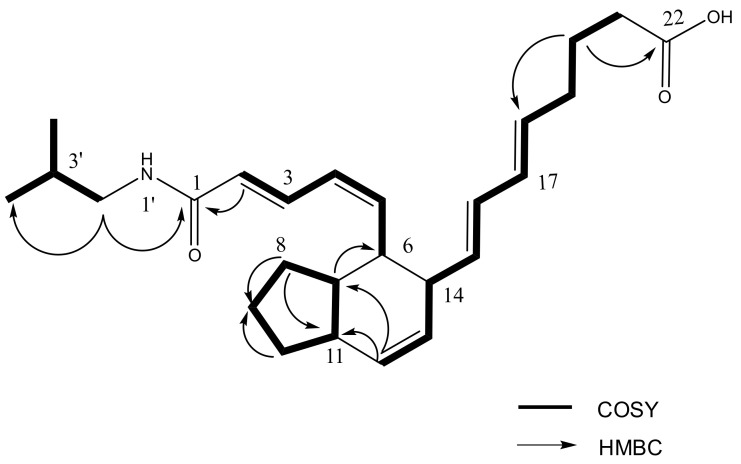
Key COSY and key HMBC correlations of **1**.

Interpretation of 2D NMR data including COSY, HSQC and HMBC spectra revealed that two rings comprise a fused bicyclic system (2, 3, 3a, 4, 5, 7a-hexahydro-4, 5-disubstituted-1H-indene) of which substituted functional groups are an octa-5, 7-dienoyl and an *N*-isobutyl 2, 4-pentadienamide moieties (Figure 2). The fused bicyclic carboxylic acids belonging to the class of aplidic acids [13] were previously reported, but aplidic acid with *N*-isobutyl 2, 4-pentadienamide moiety has not been reported. The COSY correlations between H-3′ and H-2′/H-4′/H-5′ suggested the presence of isobutyl functionality, which was also supported by the HMBC correlations from H-4′ (δ 0.92) to C-2′ (δ 48.1), C-3′ (29.8), and C-5′ (δ 20.6), and from H-5′ to C-2′, C-3′ and C-4′ (δ 20.6). Finally, the attachment of an isobutyl amide to C-1 was assigned by the HMBC correlation of H-2′ (δ 3.08) to the carbonyl carbon C-1 (δ 169.2). In particular, the geometry for the Δ*^4^* double bond of **1** was determined *Z* geometry based on the coupling constant between H-4 and H-5 (*J*_4, 5_ = 10.3 Hz).

**Figure 3:**
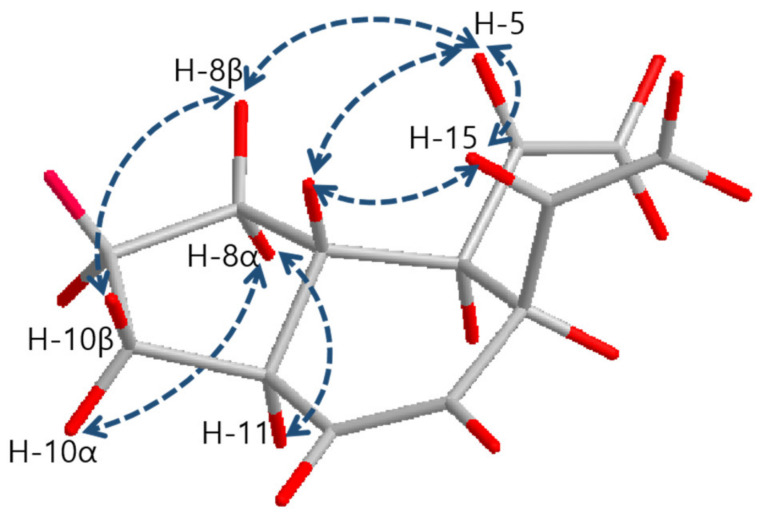
Key NOESY correlations of **1–5**.

The relative configurations of **1** were determined by the interpretation of NOESY correlations as shown in Figure 3. The NOESY correlations between H-5 (δ 5.71)/H-7 (δ 1.48), H-5 (δ 5.71)/H-15 (δ 5.54) and H-7 (δ 1.48)/H-15 (δ 5.54) provided clear evidence that explains H-5, 7 and 15 are located on the same face of the molecule. H-11 (δ 1.95) was found to be located on the opposite face against H-5, H-7 and H-15 by NOESY correlations between H-8α (δ 1.06)/H-11 (δ 1.95) and H-8β (δ 1.64)/H-5 (δ 5.71). Thus, relative stereochemistry of **1** was determined as 6R*, 7S*, 11S*, and 14R*.

#### 2.1.2. Compound **2**

The compound **2** was isolated as a yellow oil. The molecular formula of **2** was identified as C_30_H_37_O_3_N on the basis of the HRFABMS data of the [M + H]^+^ ion at *m*/*z* 460.2854 (Δ +0.8 mmu) and ^13^C NMR data (Table 2). The ^1^H and ^13^C NMR spectra of **2** are similar to those of **1** with a few notable differences which indicated that their structures were closely related each other. However, the resonances in the aromatic region (δ_H_ 7.19, δ_C_ 127.5; δ_H_ 7.23, δ_C_ 129.9; δ_H_ 7.29, δ_C_ 129.6) and the resonances of two methylene (δ_H_ 3.50, δ_C_ 42.9; δ_H_ 2.85, δ_C_ 36.7) were observed in the ^1^H and ^13^C NMR spectra of **2**, while the resonances corresponding to the isobutyl residue of 1 were absent in the ^1^H and ^13^C NMR spectra of **2**. These differences in the NMR spectra indicated the presence of a 2-phenethyl amide moiety in **2** instead of isobutyl amide as in **1**. The planar structure of **2** was elucidated as a bicyclic carboxylic acid with a 2-phenethyl amide from analysis of 1D and 2D NMR spectra for **2**, which was the same with the structure of previously reported 4*Z*-aplidic acid B [13]. Further analysis of NOESY spectra for **2** revealed that relative configurations of **2** were 6R*, 7S*, 11S*, and 14R*, the same as those of **1** at every stereogenic center.

#### 2.1.3. Compounds **3**–**5**

Compound **3** was isolated as a yellow oil. The molecular formula of **3** was identified as C_27_H_39_O_3_N on the basis of the HRFABMS data of the molecular ion peak at *m*/*z* 426.3005 (Δ + 0.2 mmu) and ^13^C NMR data (Table 2). Fourteen mass units from that of compound **1** and the similar pattern of ^1^H NMR spectrum of **1** and **3** suggested that **3** is an analogue of **1** with one more methyl or methylene. COSY correlations between H-2′(δ 3.07, 3.19)/H-3′(δ 1.60), H-3′/H-6′(δ 0.93), H-3′/H-4′(δ 1.44, 1.17) and H-4′/H-5′(δ 0.91) revealed that isobutyl amide residue of **1** is replaced with 2-methylbutyl amide in **3** and the planar structure of **3** was elucidated as a fused bicyclic carboxylic acid with octa-5, 7-dienoyl and an *N*-(2-methylbutyl)-2, 4-pentadienamide moieties.

Compound **4** was isolated as a yellow oil. The molecular formula of **4** was identified as C_23_H_30_O_4_ on the basis of the HRFABMS data of the [M + H]^+^ ion at *m*/*z* 371.2209 (Δ − 0.8 mmu) and ^13^C NMR data (Table 2). Interpretation of the spectroscopic data of **4** revealed that the planar structure of **4** is a bicyclic fatty acid with octa-5, 7-dienoyl and methyl penta-2,4-dienoate moieties, the same with a previous reported fatty acid derivative, aplidic acid A [13] except for the geometry of the double bond between C-4 and C-5. The geometry for the Δ^4^ double bond was determined *Z* geometry on the basis of the coupling constant between H-4 and H-5 (*J*_4, 5_ = 11.0 Hz).

Compound **5** was isolated as a yellow oil. The molecular formula of **5** was identified as C_22_H_28_O_4_ on the basis of the HRFABMS data of the [M + Na]^+^ ion at *m*/*z* 379.1884 (Δ + 0.4 mmu) and ^13^C NMR data (Table 2). The similarity of ^1^H NMR spectrum of **5** to that of **4** and the difference of 14 mass units in HRMS suggested that the structures of **5** and **4** could be closely related with a slight difference of one less methyl or methylene. The distinguished methoxy signal in the ^1^H NMR spectrum of **4** disappeared in the ^1^H NMR spectrum of **5**. The inspection of the spectroscopic data of **5** led to the planar structure of **5** as a fused bicyclic fatty acid with octa-5, 7-dienoyl and penta-2, 4-dienoate moieties.

The relative configurations of **3**–**5** were determined as same with **1** and **2** as 6S*, 7R*, 11R*, and 14S* on the basis of the common NOESY correlations for **1**–**5** between H-5/H-7, H-5/H-15 and H-7/H-15 (Figure 3).

#### 2.1.4. The Absolute Configurations for **1**–**5**

**Figure 4:**
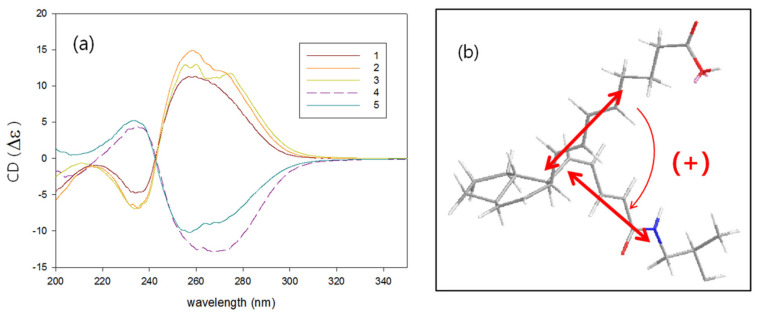
(**a**) CD spectra of **1**–**5**. (**b**) Sign of exciton chirality of **1**.

Surprisingly, the amide compounds **1**–**3** had positive sign of specific rotation while carboxylic compounds **4** and **5** had negative in spite of the same relative configurations for all compounds. A dramatic difference in chiroptical properties between amides and carboxylic compounds also appeared in the CD spectra of **1**–**5**. Amides **1**–**3** showed completely split circular dichroism curve; positive Cotton effect at *λ* 260 nm and negative at *λ* 234 nm, while carboxylic compounds **4** and **5** showed negative Cotton effect at the longer and positive at the shorter wavelength (Figure 4a). On the basis of these exciton-coupled circular dichroism data and energy-minimized structure modeling, absolute configurations for **1**–**5** were determined [25]. The absolute configurations of **1** were determined as 6*S*, 7*R*, 11*R*, and 14*S* by a positive CD spectrum which is originated from the positive helicity of the diene chromophores (Figure 4b). The absolute configuration of **2** and **3** was determined as the same with those of **1** (6*S*, 7*R*, 11*R*, 14*S*) from the CD absorptions, while the absolute configuration of **4** and **5** was determined as 6*R*, 7*S*, 11*S*, and 14*R* from the bisignate CD pattern attributable with the negative helicity.

The structures of **1**–**5** were very similar to the bicyclic fatty acid derivatives isolated from colonial tunicates of the family Polyclinidae [12,13]. Especially, **2** and **4** were stereoisomers of 4*Z*-aplidic acid B and aplidic acid A, respectively, reported by Bao et al. [13]. Although the source organisms bearing the two similar series of molecules do not even belong to the same family, their ways of life are very similar; sedentary, filter-feeding, and colonial growth. The differences between the two similar series of molecules in stereochemistry at key stereogenic centers are hypothesized to arise from the different type of Diels–Alder cyclization as proposed by Bao et al. [13]. The structures of compounds **1** and **3** were reported for the first time, as their amino acid parts condensated with a carboxylate group were different with previous reported metabolites [13].

As natural products with the same skeletons are usually biosynthesized via the same biosynthetic pathways engaged by the same enzymes, they share the same absolute configurations in general. However, in this case, five fatty acid derivatives sharing the same carbon framework exhibited contrary Cotton effects, which led to the assignment of the enantiomers. The contrast was dependent on the possession of amino acid adducts. Previously, didemnones [26], another class of fatty acid derivatives isolated from colonial tunicates were reported as an example of natural products with enantiomeric series. In addition, Perry and Weavers reported isolation and biosynthesis of enantiomeric series of diterpenes [27]. Our discovery of enantiomeric natural products is another rare example of natural products biosynthesis, but it is explainable by the biogenetic hypothesis of the key intramolecular cyclization in the Diels–Alder manner.

#### 2.1.5. Spectroscopic Data of Compounds **1**–**5**

Compound **1**: yellow oil; [α]25D +103 (c 0.005, MeOH); UV (MeOH) *λ*_max_ (log ε) 266 (4.08), 239(4.13) nm; CD (*c* 2.4 × 10^−3^ M, MeOH) *λ*_max_ (Δε) 260 (+11.3), 234 (−4.8) nm; IR (KBr) *ν*_max_ 3335, 2959, 1714, 1659, 1261 cm^−1^; ^1^H and ^13^C NMR data, see Table 1; HRFABMS *m*/*z* 412.2850 [M + H]^+^ (calcd for C_26_H_38_O_3_N^+^, 412.2846).

Compound **2**: yellow oil; [α]25D +196 (c 0.005, MeOH); UV (MeOH) *λ*_max_ (log ε) 264 (4.09), 239(4.13) nm; CD (*c* 4.1 × 10^−3^ M, MeOH) *λ*_max_ (Δε) 259 (+14.9), 235 (−6.8) nm; IR (KBr) *ν*_max_ 3335, 2959, 1714, 1659, 1261 cm^−1^; ^1^H and ^13^C NMR data, see Table 2; HRFABMS *m*/*z* 460.2854 [M + H]^+^ (calcd for C_30_H_38_O_3_N^+^, 460.2846).

Compound **3**: yellow oil; [α]25D +154 (c 0.005, MeOH); UV (MeOH) *λ*_max_ (log ε) 263 (4.25) 239(4.22) nm; CD (*c* 2.9 × 10^−3^ M, MeOH) *λ*_max_ (Δε) 260 (+13.0) 234(−6.9) nm; IR (KBr) *ν*_max_ 3335, 2959, 1714, 1659, 1261 cm^−1^; ^1^H and ^13^C NMR data, see Table 2; HRFABMS *m*/*z* 426.3005 [M + H]^+^ (calcd for C_27_H_40_O_3_N^+^, 426.3003).

Compound **4**: yellow oil; [α]25D −101 (c 0.005, MeOH); UV (MeOH) *λ*_max_ (log ε) 265 (4.30), 239(4.24) nm; CD (*c* 2.0 × 10-3 M, MeOH) *λ*_max_ (Δε) 267 (−12.8), 235( +4.4) nm; IR (KBr) *ν*_max_ 3335, 2959, 1714, 1659, 1261 cm^−1^; ^1^H and ^13^C NMR data, see Table 2; HRFABMS *m*/*z* 371.2209 [M + H]^+^ (calcd for C_23_H_31_O_4_^+^, 371.2217).

Compound **5**: yellow oil; [α]25D −92 (c 0.005, MeOH); UV (MeOH) *λ*_max_ (log ε) 261 (4.33), 239(4.33) nm; CD (*c* 2.0 × 10-3 M, MeOH) *λ*_max_ (Δε) 257 (−10.2), 233( +5.2) nm; IR (KBr) *ν*_max_ 3335, 2959, 1714, 1659, 1261 cm^−1^; ^1^H and ^13^C NMR data, see Table 2; HRFABMS *m*/*z* 379.1884 [M + Na]^+^ (calcd for C_22_H_28_O_4_Na^+^, 379.1880).

### 2.2. Antibacterial Activity of the Compounds

Compounds **1**–**4** were tested for antibacterial activity against *Staphylococcus aureus* (CCARM 0204). Compounds **1** and **2** exhibited inhibitory activity against the pathogen with the minimum inhibitory concentration (MIC) of 16 and 2 μg/mL, respectively (MIC (μg/mL) of positive controls: vancomycin (0.1); linezolid (3.2); daptomycin (3.2)). In addition, Jung’s group reported a series of cytotoxic analogues having the same bicyclic moiety and similar side chains from a tunicate of the family Polyclinidae [13]. These observations suggest that these bicyclic fatty acids do not act as ordinary primary metabolites and may play an important role in the chemical defenses evolved by tunicates.

## 3. Materials and Methods

### 3.1. General Experimental Procedures

Optical rotations were measured in MeOH using a 1.0 cm cell on a Rudolph Research (Autopol III, Hackettstown, NJ, USA). UV spectra were also recorded in MeOH on a Scinco UVS-2100 (Seoul, Korea). CD spectra were taken in MeOH in an Applied Photophysics Chirascan plus (Leatherhead, UK). IR spectra were recorded on KBr plates with a Thermo Nicolet 570 (Waltham, MA, USA). NMR spectra were recorded on a Bruker Avance DPX-600 (Billerica, MA, USA) and Bruker Ascend 700 spectrometer using MeOD as solvent. High resolution mass spectra were acquired on a JEOL, JMS-AX505WA mass spectrometer (Tokyo, Japan).

### 3.2. Animal Material

A species of dark purple colonial tunicate was collected by scuba in the South Sea of Korea. The sample was frozen immediately after collection Voucher specimen (CMDD11B0309) was anesthetized with 5% menthol in sterilized seawater for 2 h and stored 10% formalin in sterilized seawater. The animal was taxonomically identified by one of the authors (B.J.R.). The voucher was deposited at the Ewha Womans University Natural History Museum, Korea and at the Center for Marine Natural Products and Drug Discovery, Seoul National University, Korea. The tunicate was identified as *Didemnum* sp.

### 3.3. Extraction and Isolation of Compounds

The frozen animal (600 g, wet wt.) was lyophilized, and the dried specimen (120 g) was extracted thrice with 50% MeOH in DCM. The extract (30 g) was dried in vacuo. The dried extract was dissolved in DCM and then washed twice with distilled water. After removal of the solvent, the DCM-soluble fraction (10.0 g) was partitioned to 90% aqueous MeOH and hexane fractions. The aqueous MeOH-soluble fraction (4.0 g) was further separated into 13 fractions by Sephadex^®^ LH-20 open column chromatography using 50% MeOH in DCM as the eluent. Each fraction was tested for antibacterial activity against bacterial strains, and fractions #8 and #10 showed antibacterial activity against Gram positive bacterial strains (Staphylococcus aureus CCARM0204 and S. aureus CCARM 0205). Fractions #8 and #10 were independently subjected to reversed-phase HPLC (Phenomenex Luna C18 (2), 5 μm, 100 Å, 250 × 100 mm, Torrance, CA, USA) monitoring UV absorption at 230 nm, using a gradient mobile phase mixture of acetonitrile and water at a flow rate of 2.0 mL/min, to afford **1**, **2**, **3** and **4**. By comparisons of spectroscopic data (^1^H NMR and LC-MS) of fraction #12 with bioactive fractions, fraction #12 was assumed to have another derivative, so that **5** was isolated using the same reversed-phase HPLC isolation scheme with **1**–**3** and **4**. The amount of compounds **1**–**5** was 7.1, 9.2, 5.5, 4.3, and 0.8 mg, respectively.

### 3.4. Bioassay Procedures

The following pathogen, obtained from the stock Culture Collection of Antimicrobial Resistant Microorganisms (Seoul Women’s University, Seoul, Korea), was used in this bioassay study: *Staphylococcus aureus* CCARM 0204. The antibacterial activity was determined by the 2-fold microtiter broth dilution method [28]. Doubling dilutions of tested compounds were added to each well of a 96-well microtiter plate containing a fixed volume of Mueller Hinton II broth (Difco, Franklin Lakes, NJ, USA) (final 0.64% DMSO; the concentrations tested: 64~0.125 µg/mL). The positive control (vancomycin, linezolid and daptomycin) was tested as well and checked its MIC within one twofold dilution of published values for the quality control. Each well was inoculated with an overnight culture of bacteria (5 × 10^5^ CFU/mL) and incubated at 37 °C for 24 h. The minimum inhibitory concentration (MIC) was taken as the lowest concentration of the tested compounds, at which no growth was observed with the unaided eye.

## Figures and Tables

**Table 1:** NMR spectroscopic data for compound **1**^a^.

No.	*δ*_C_, mult.	*δ*_H_, mult., (*J* in Hz)	COSY	HMBC
**1**	169.2, C			
**2**	125.4, CH	6.02, d (15.0)	3	1, 4
**3**	137.0, CH	7.55, dd ( 15.0, 11.7)	2, 4	1,2, 4, 5
**4**	127.5, CH	6.17, dd ( 11.7, 10.3)	3, 5	2, 3, 6
**5**	143.0, CH	5.71, dd (10.3, 10.3)	4, 6	3, 7
**6**	44.2, CH	3.02, m	5, 7	4, 7
**7**	45.1, CH	1.48, m	6, 8, 11	6,8, 11
**8**	29.0, CH_2_	1.06 α, 1.64 β, m	7, 9	7,9,10,11
**9**	23.1, CH_2_	1.71, m	8, 10	
**10**	30.3, CH_2_	1.89 α, 1.21 β, m	9, 11	7,8, 9, 11
**11**	46.8, CH	1.95, m	7, 10, 12	
**12**	131.0, CH	5.92, d (10.0)	11, 13	7, 11
**13**	130.9, CH	5.46, ddd(10.0, 3.3,3.3)	12, 14	11
**14**	46.7, CH	3.02, m	13, 15, 16	12, 13, 16
**15**	133.0, CH	5.54, dd (15.0, 7.8)	14, 16	14, 17
**16**	133.5, CH	5.94, dd (15.0, 10.3)	14, 15, 17	14, 18
**17**	132.3, CH	6.04, dd (15.0, 10.3)	16, 18	18, 19
**18**	133.2, CH	5.57, dd (15.0, 7.2)	17, 19	16, 19, 20
**19**	33.1, CH_2_	2.11, td (7.2, 7.2)	17, 18, 20	18, 20, 21
**20**	25.9, CH_2_	1.68, m	19, 21	18,19, 21, 22
**21**	34.5, CH_2_	2.28, t (7.4)	20	19, 20, 22
**22**	177.7, C			
**1′**	NH			
**2′**	48.1, CH_2_	3.08, d (6.9)	3′	1, 3′, 4′
**3′**	29.8, CH	1.81, m	2′, 4′	2′, 4′
**4′**	20.6, CH_3_	0.92, d (6.7)	3′	2′, 3′, 5′
**5′**	20.6, CH_3_	0.92, d (6.7)	3′	2′, 3′, 4′

^a 1^H NMR data were measured at 600 MHz and ^13^C NMR data were measured at 150 MHz in methanol-*d*_4_.

**Table 2:** ^1^H and ^13^C NMR spectroscopic data for **2**–**5**^a^.

=	2		3		4		5	
No.	δ_C_, mult.	δ_H_, mult., (*J* in Hz)	δ_C_, mult.	δ_H_, mult., (*J* in Hz)	δ_C_, mult.	δ_H_, mult., (*J* in Hz)	^b^ δ_C_, mult.	δ_H_, mult., (*J* in Hz)
**1**	169.2, C		169.0, C		169.6, C		173.9, C	
**2**	125.9, CH	5.97, d(15.1)	125.4, CH	6.02, d(15.2)	122.1, CH	5.92, d(15.2)	123.0, CH	5.89, d(15.0)
**3**	137.1, CH	7.55, dd(15.1, 11.7)	136.9, CH	7.54, dd(15.2, 11.7)	141.4, CH	7.66, dd(15.2, 11.7)	141.5, CH	7.61, dd(15.0, 11.7)
**4**	127.5, CH	6.17,dd(11.7, 11.0)	127.4, CH	6.16, dd(11.7, 11.0)	127.3, CH	6.20, dd(11.7, 11.0)	127.1, CH	6.19, dd(11.7, 11.0)
**5**	143.2, CH	5.73, dd(11.0, 10.5)	143.0, CH	5.71, dd(11.0, 10.3)	145.2, CH	5.81, dd(11.0, 10.5)	144.4, CH	5.76, dd(11.0, 10.5)
**6**	44.2, CH	3.04, m	44.1, CH	3.03, m	44.4, CH	3.01, m	44.1, CH	3.00, m
**7**	45.2, CH	1.49, m	45.1, CH	1.48, m	45.1, CH	1.49, m	45.0, CH	1.45, m
**8**	29.1, CH_2_	1.08α, 1.69β, m	29.0, CH_2_	1.07α,1.63β, m	29.2, CH_2_	1.04α, 1.64β, m	29.1, CH_2_	1.03α, 1.62β, m
**9**	23.1, CH_2_	1.73, m	23.1, CH_2_	1.71, m	23.1, CH_2_	1.72, m	23.0, CH_2_	1.70, m
**10**	30.3, CH_2_	1.91α, 1.23β, m	30.4, CH_2_	1.89α, 1.21β, m	30.4, CH_2_	1.89α, 1.22β, m	30.3, CH_2_	1.87α, 1.24β, m
**11**	46.9, CH	1.97, m	46.8, CH	1.95, m	46.8, CH	1.95, m	46.6, CH	1.93, m
**12**	131.1, CH	5.95, d(10.0)	131.0, CH	5.93, d(10.0)	131.1, CH	5.93, d(10.0)	130.9, CH	5.91, d(10.0)
**13**	131.0, CH	5.48, ddd(10.0, 3.3, 3.3)	130.9, CH	5.46, ddd(10.0, 3.3, 3.3)	130.9, CH	5.47, ddd(9.9, 3.3, 3.3)	130.8, CH	5.45, ddd(10.0, 3.3, 3.3)
**14**	46.8, CH	3.04, m	46.7 CH	3.03, m	46.8, CH	3.01, m	46.7, CH	3.00, m
**15**	133.0, CH	5.55, dd(15.0, 7.7)	133.0, CH	5.55, dd(15.0, 7.7)	132.8, CH	5.54, dd(15.0, 7.8)	132.8, CH	5.53, dd(15.0, 7.7)
**16**	133.6, CH	5.97, dd(15.0, 10.3)	133.5, CH	5.95, dd(15.0, 10.3)	133.7, CH	5.95, dd(15.0, 10.3)	133.4, CH	5.93, dd(15.0, 10.3)
**17**	132.4, CH	6.05, dd(15.0, 10.3	132.4, CH	6.04, dd(15.0, 10.3	132.4, CH	6.04, dd(15.0, 10.3)	132.2, CH	6.03, dd(15.0, 10.3)
**18**	133.3, CH	5.59, dd(15.0, 7.2)	133.2, CH	5.56, dd(15.0, 7.2)	133.4, CH	5.57, dd(15.0, 7.2)	133.3, CH	5.56, dd(15.0, 7.2)
**19**	33.2, CH_2_	2.12, td(7.2, 7.2)	33.2, CH_2_	2.11, td(7.2, 7.2)	33.1, CH_2_	2.10, td(7.2, 7.2)	33.1, CH_2_	2.08, td(7.2, 7.2)
**20**	26.1, CH_2_	1.70, m	25.0, CH_2_	1.68, m	26.0, CH_2_	1.68, m	26.0, CH_2_	1.66, m
**21**	34.7, CH_2_	2.29, t(7.4)	34.7, CH_2_	2.28, t(7.4)	34.5, CH_2_	2.28, t(7.4)	34.5, CH_2_	2.28, t(7.4)
**22**	177.9, C		177.8, C		177.7, C		178.0, C	
**1′**	NH		NH					
**2′**	42.9, CH_2_	3.50, t(7.4)	46.4, CH_2_	3.07, dd(13.3, 6.2)3.19, dd(13.3, 6.3)				
**3′**	36.7, CH_2_	2.85, t(7.4)	38.4, CH	1.60, m				
**4′**	140.7, C		28.3, CH_2_	1.44, 1.17, m				
**5′**	129.9, CH	7.23, dd(7.0, 1.1)	17.7, CH_3_	0.91, t(6.8)				
**6′**	129.6, CH	7.29	11.7, CH_3_	0.93, d(6.6)				
**7′**	127.5, CH	7.19, ddd(7.3, 7.3, 1.1)						
**8′**	129.6, CH	7.29, ddd(7.3, 7.0, 1.1)						
**9′**	129.9, CH	7.23, dd(7.0, 1.1)						
OCH_3_					52.2, OCH_3_	3.74, s		

^a 1^H NMR data were measured at 600 MHz and ^13^C NMR data were measured at 150 MHz in methanol-*d*_4_. ^b^ The chemical shifts for the quaternary carbons of **5** were acquired from signals in the HMBC spectrum.

## Supplementary Materials

The following are available online at https://www.mdpi.com/article/10.3390/md19090521/s1, Figure S1. ^1^H NMR spectrum of compound **1** in methanol-*d*4. Figure S2. ^13^C NMR spectrum of compound **1** in methanol-*d*4. Figure S3. COSY spectrum of compound **1** in methanol-*d*4. Figure S4. HSQC spectrum of compound **1** in methanol-*d*4. Figure S5. HMBC spectrum of compound **1** in methanol-*d*4. Figure S6. NOESY spectrum of compound **1** in methanol-*d*4. Figure S7. ^1^H NMR spectrum of compound **2** in methanol-*d*4. Figure S8. ^13^C NMR spectrum of compound **2** in methanol-*d*4. Figure S9. ^1^H NMR spectrum of compound **3** in methanol-*d*4. Figure S10. ^13^C NMR spectrum of compound **3** in methanol-*d*4. Figure S11. ^1^H NMR spectrum of compound **4** in methanol-*d*4. Figure S12. ^13^C NMR spectrum of compound **4** in methanol-*d*4. Figure S13. ^1^H NMR spectrum of compound **5** in methanol-*d*4.
