# Supplementary material for: Antibacterial Bicyclic Fatty Acids from a Korean Colonial Tunicate Didemnum sp
Source: Mar Drugs. 2021 Sep 16;19(9):521. doi: 10.3390/md19090521 (PMC8465582; doi:10.3390/md19090521)
Supplement: Supplementary file 1 [file marinedrugs-19-00521-s001.zip › marinedrugs-1380578-supplementary.pdf]

Supplementary Materials

Antibacterial Bicyclic Fatty Acids from a Korean Colonial Tunicate *Didemnum* sp.

Hiyoung Kim[1,2], Jusung Lee[2], Tae Gu Lee[3], Inho Yang[4], Jungwook Chin[5], Boon Jo Rho[6], Hyukjae Choi[7], Sang-Jip Nam[8], Dongyup Hahn[9,10,*] and Heonjoong Kang [2,10,11,*]

[1] Department of Biomedical Science and Engineering, Konkuk University, Seoul 05029, Korea
[2] Laboratory of Marine Drugs, School of Earth and Environmental Sciences, Seoul National University, NS-80, Seoul 08826, Korea
[3] Safety Research Team, Crop Protection Research Institute, FarmHannong Co., Ltd, Nonsan 33010, Korea
[4] Department of Convergence Study on the Ocean Science and Technology, Korea Maritime and Ocean University, Busan 49112, Korea
[5] New Drug Development Center, Daegu-Gyeongbuk Medical Innovation Foundation, Daegu, 41061, Korea
[6] Natural History Museum, Ewha Womans University, Seoul 03760, Korea
[7] College of Pharmacy, Yeungnam University, Gyeongsan 38541, Korea
[8] Department of Chemistry and Nano Science, Ewha Womans University, Seoul 03760, Korea
[9] School of Food Science and Biotechnology & Department of Integrative Biology, Kyungpook National University, Daegu 41566, Korea
[10] Interdisciplinary Graduate Program in Genetic Engineering, Seoul National University, NS-80, Seoul 08826, Korea
[11] Research Institute of Oceanography, Seoul National University, Seoul 08826, Korea
* Correspondence: dohahn@knu.ac.kr; Tel.: +82-53-950-5756 (D.H.)
hjkang@snu.ac.kr; Tel.: +82-2-880-5730 (H.K.)

Table of Contents

Figure S1. ^{1}H NMR spectrum of compound **1** in methanol-*d*4

Figure S2. ^{13}C NMR spectrum of compound **1** in methanol-*d*4

Figure S3. COSY spectrum of compound **1** in methanol-*d*4

Figure S4. HSQC spectrum of compound **1** in methanol-*d*4

Figure S5. HMBC spectrum of compound **1** in methanol-*d*4

Figure S6. NOESY spectrum of compound **1** in methanol-*d*4

Figure S7. ^{1}H NMR spectrum of compound **2** in methanol-*d*4

Figure S8. ^{13}C NMR spectrum of compound **2** in methanol-*d*4

Figure S9. ^{1}H NMR spectrum of compound **3** in methanol-*d*4

Figure S10. ^{13}C NMR spectrum of compound **3** in methanol-*d*4

Figure S11. ^{1}H NMR spectrum of compound **4** in methanol-*d*4

Figure S12. ^{13}C NMR spectrum of compound **4** in methanol-*d*4

Figure S13. ^{1}H NMR spectrum of compound **5** in methanol-*d*4

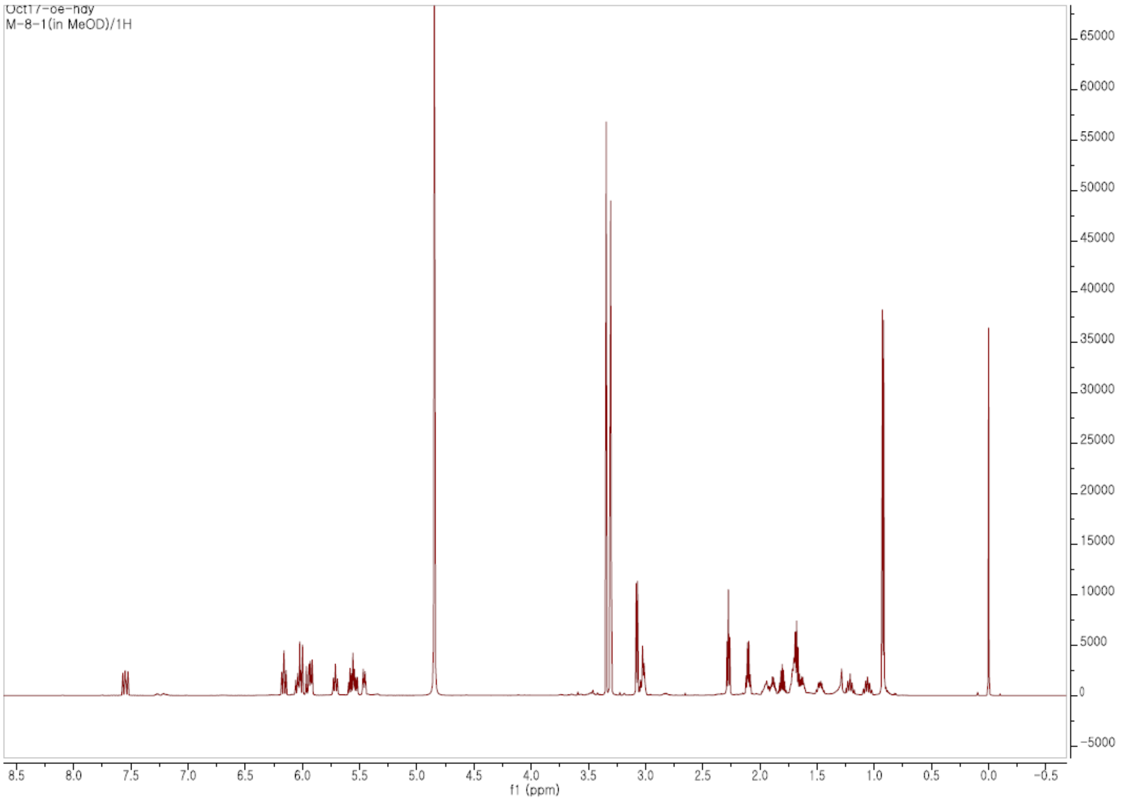

Figure S1. ^{1}H NMR spectrum of compound **1** in methanol-d_4

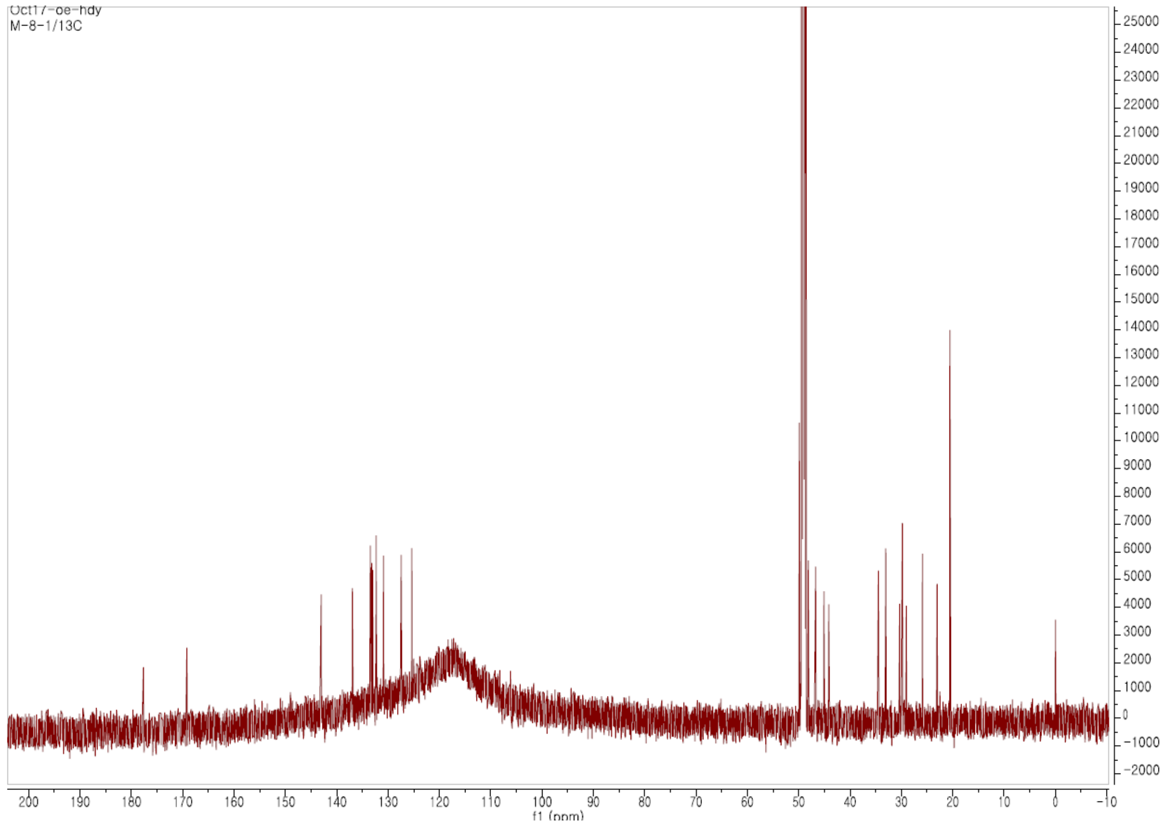

Figure S2. ^{13}C NMR spectrum of compound **1** in methanol-d_4

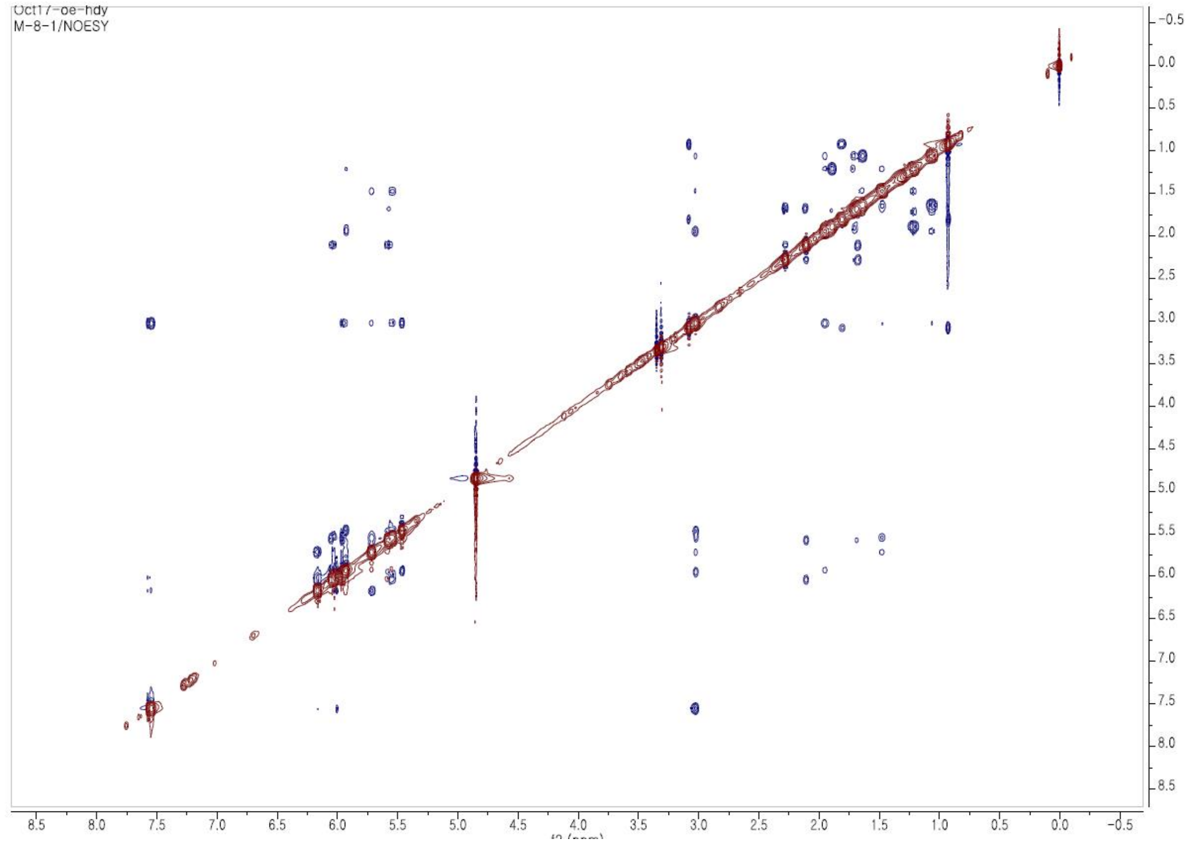

Figure S3. COSY spectrum of compound **1** in methanol-*d*4

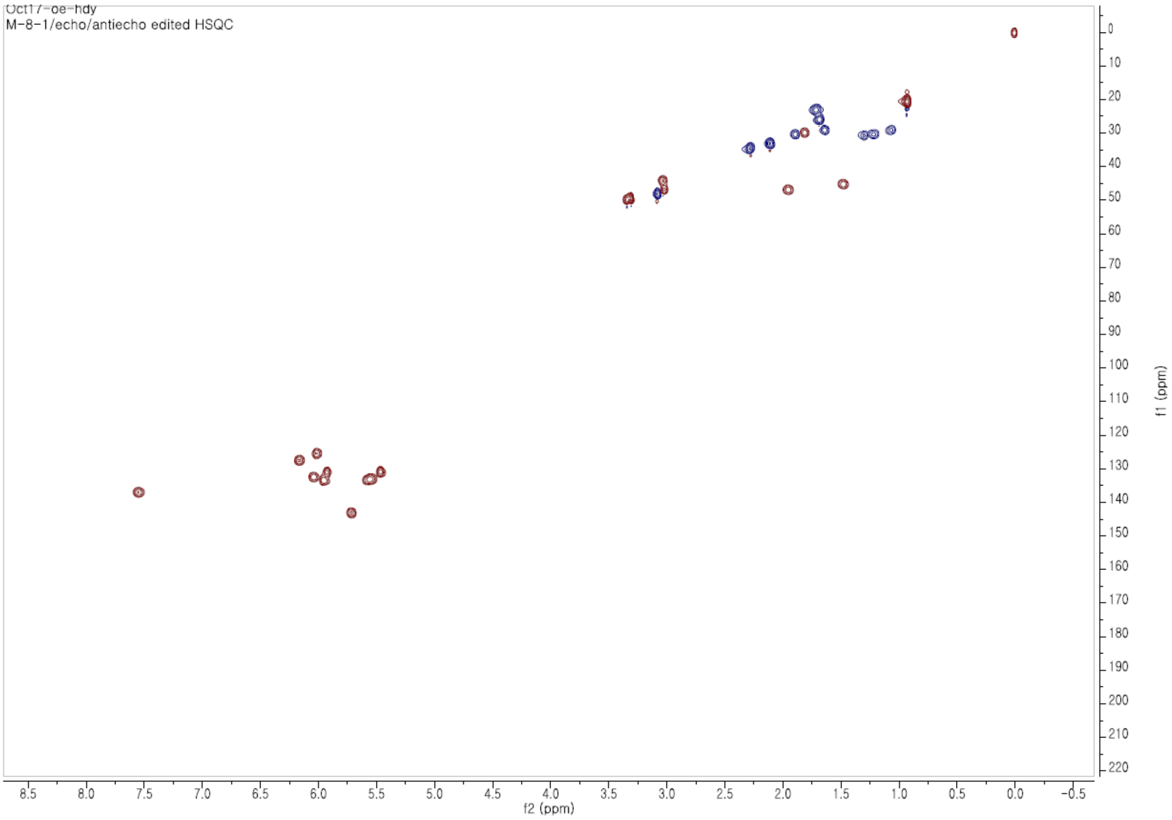

Figure S4. HSQC spectrum of compound **1** in methanol-*d*4

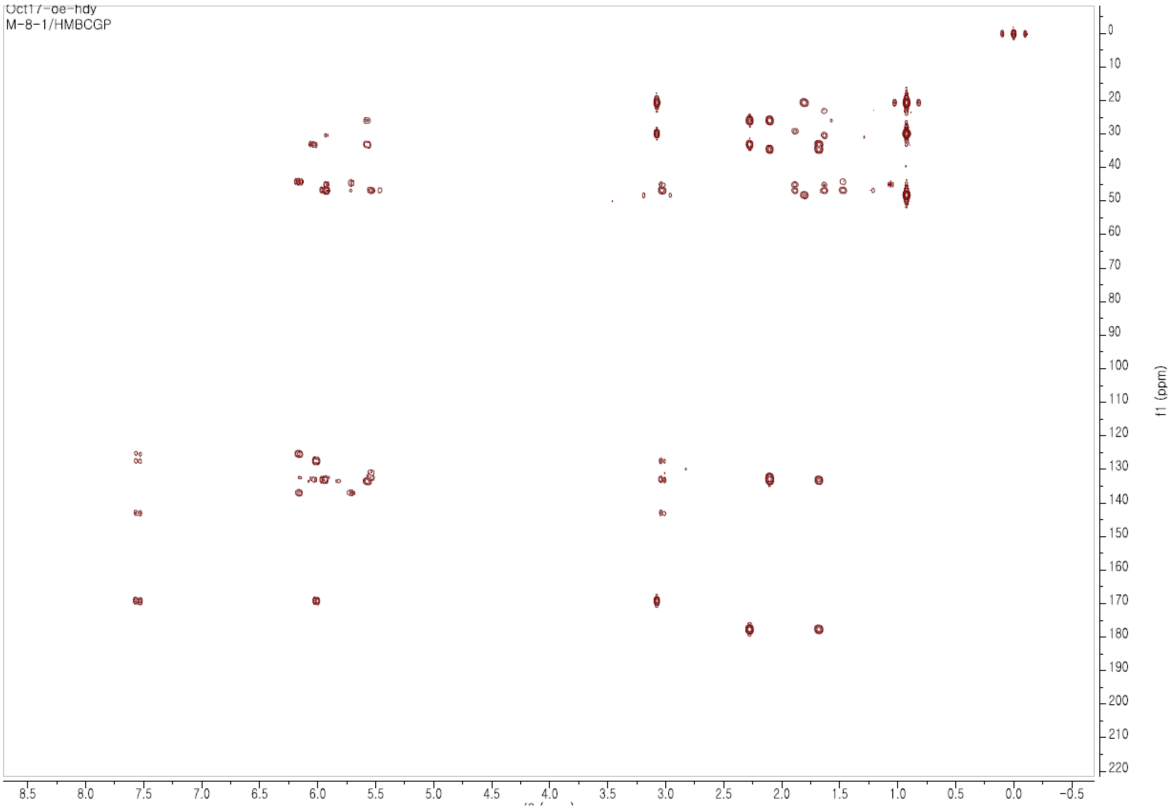

Figure S5. HMBC spectrum of compound **1** in methanol-*d*4

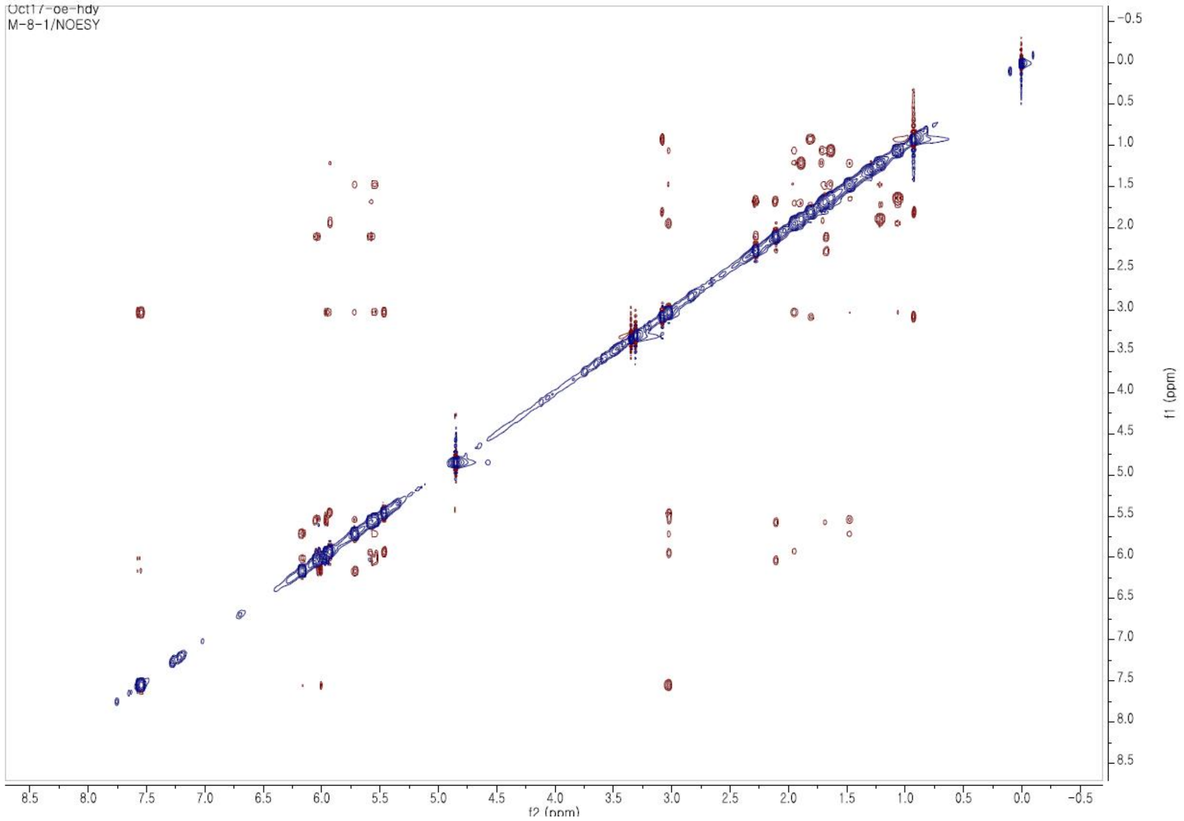

Figure S6. NOESY spectrum of compound **1** in methanol-d_4

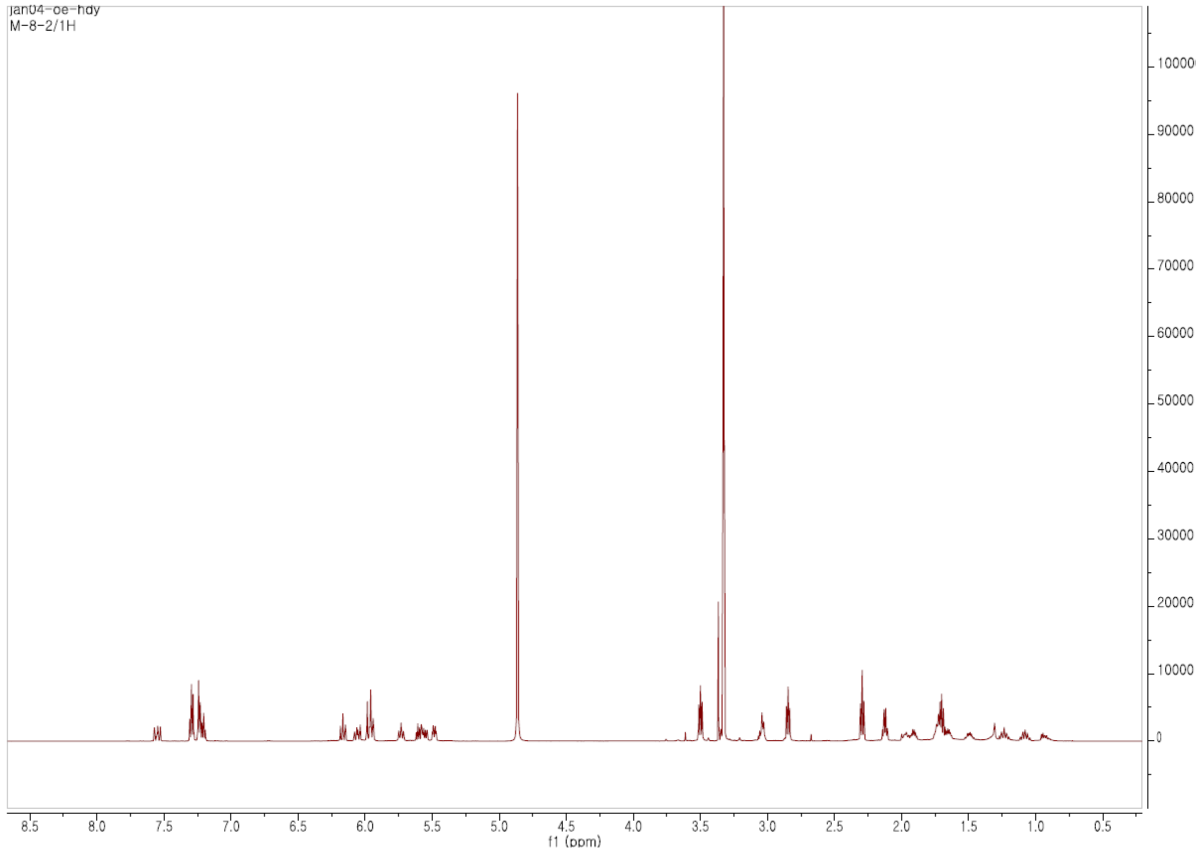

Figure S7. ^{1}H NMR spectrum of compound **2** in methanol-*d*4

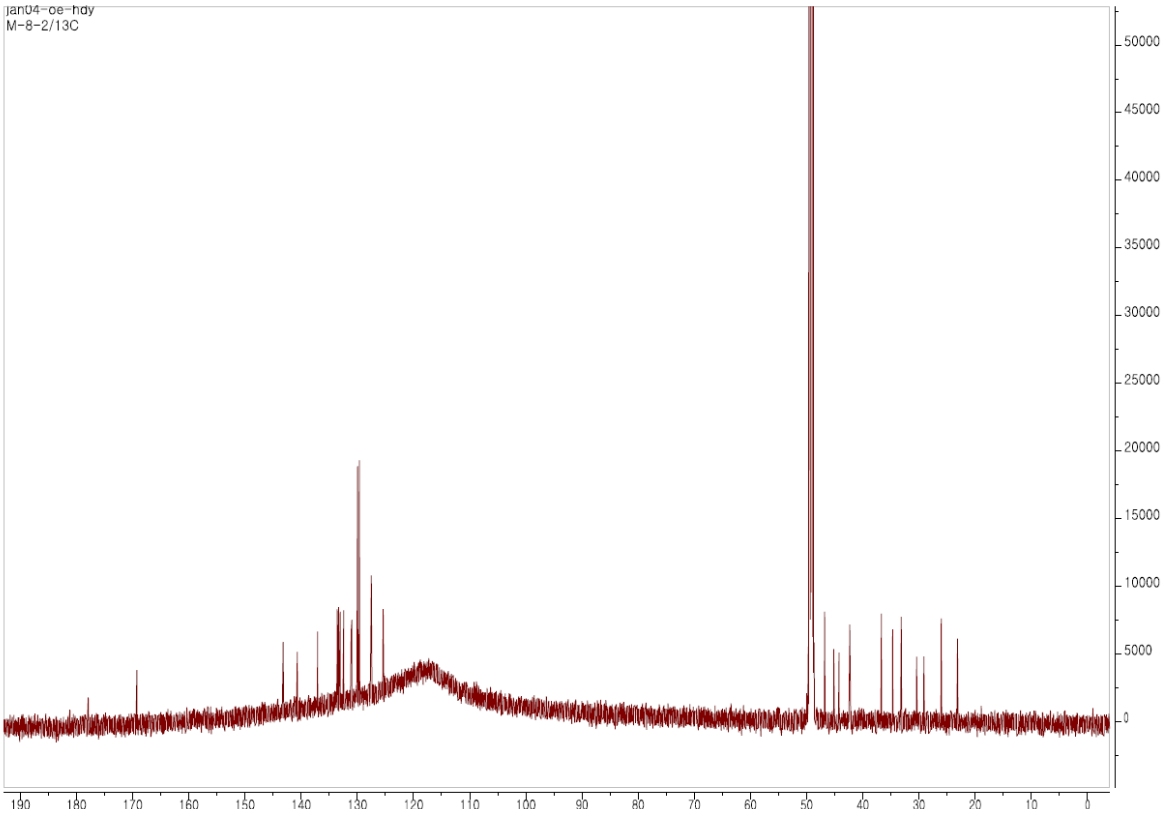

Figure S8. ^{13}C NMR spectrum of compound **2** in methanol-d_4

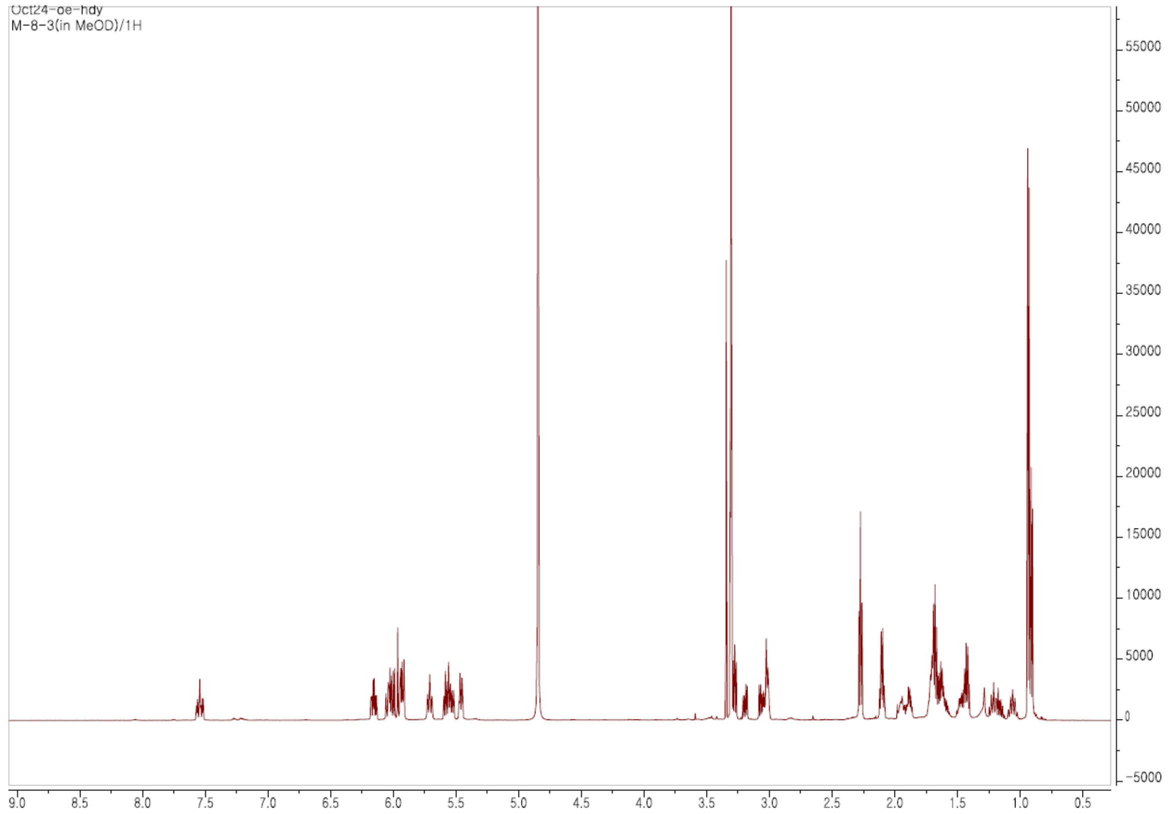

Figure S9. ^{1}H NMR spectrum of compound **3** in methanol-*d*4

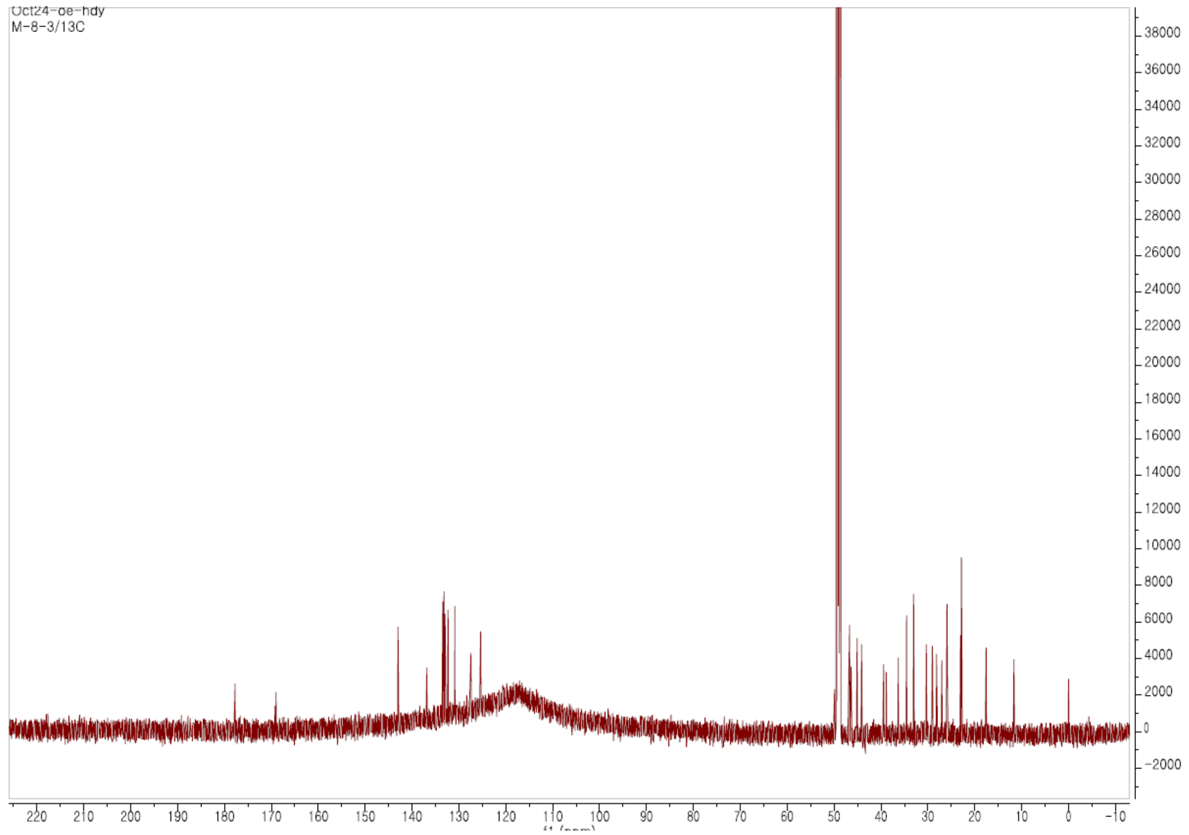

Figure S10. ^{13}C NMR spectrum of compound **3** in methanol-d_4

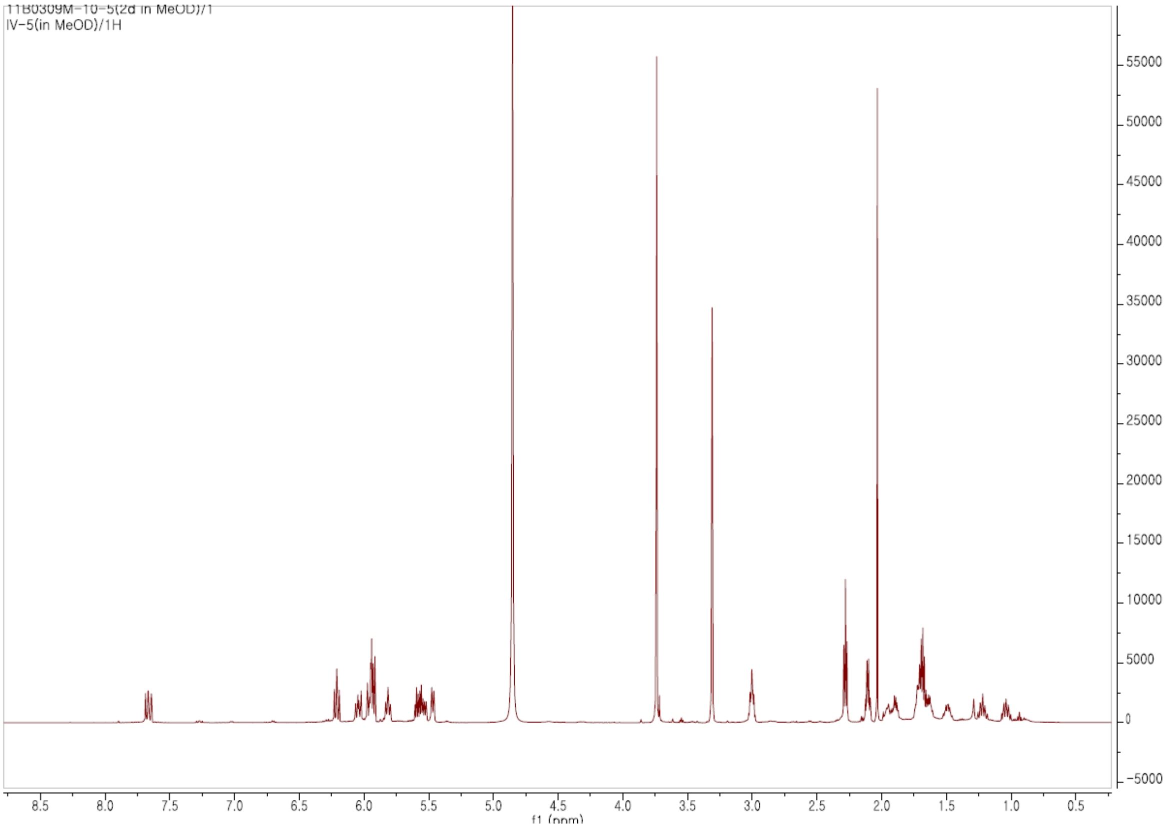

Figure S11. ^{1}H NMR spectrum of compound **4** in methanol-*d*4

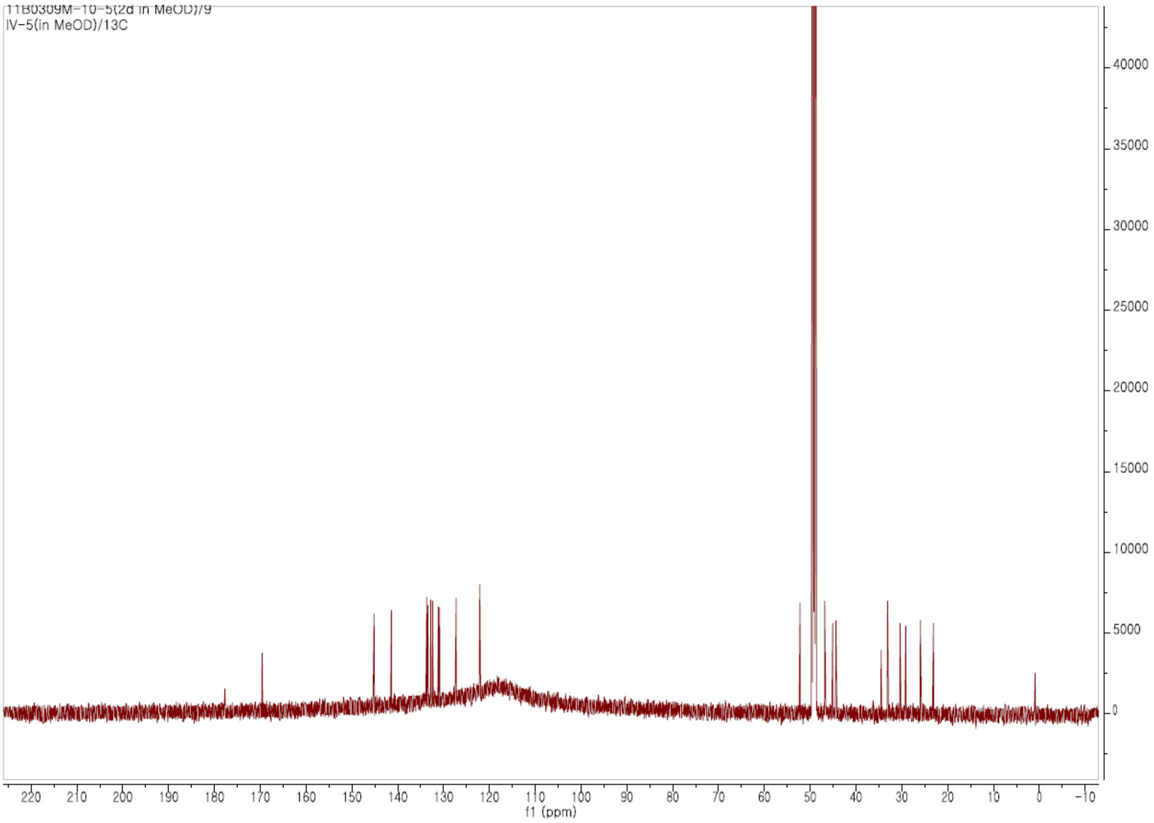

Figure S12. ^{13}C NMR spectrum of compound **4** in methanol-d_4

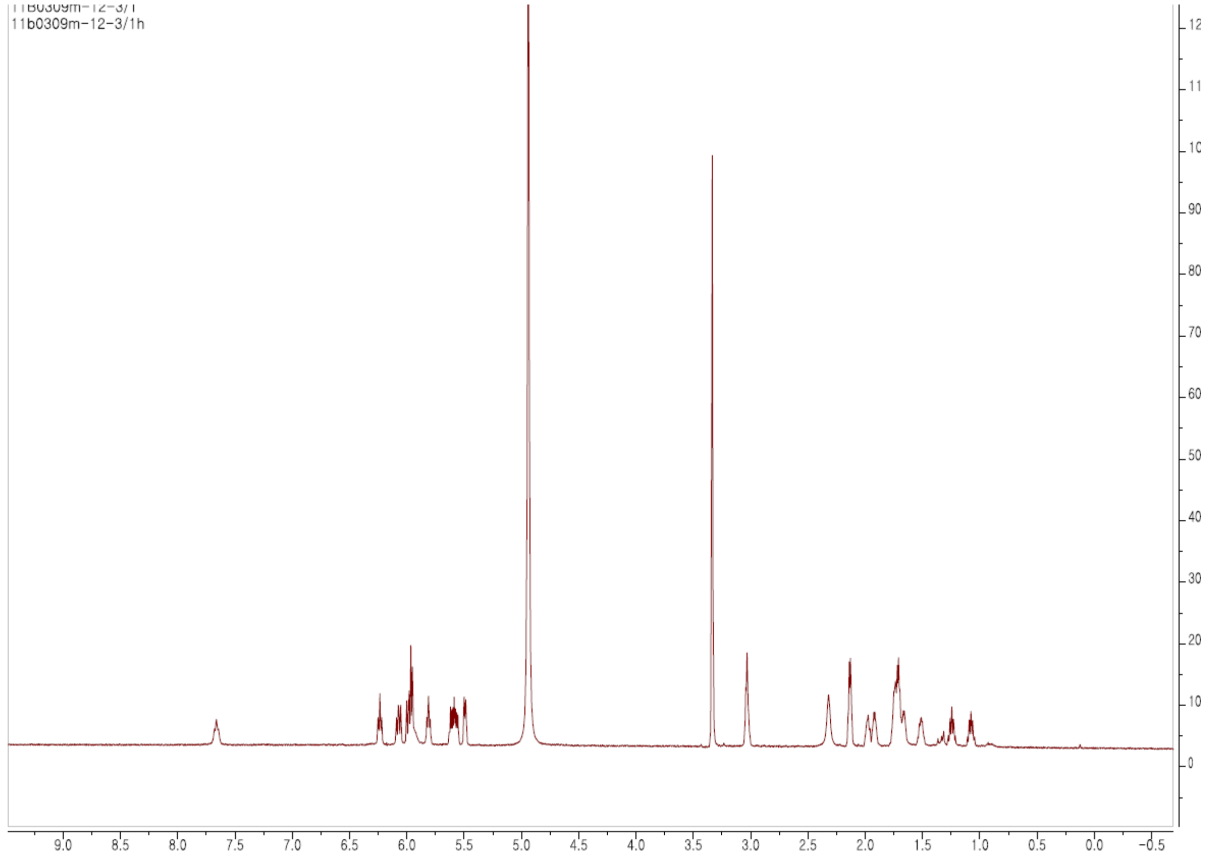

Figure S13. ^{1}H NMR spectrum of compound **5** in methanol-*d*4
